# Children’s Abrasions in Recreational Beach Areas and a Review of Possible Wound Infections

**DOI:** 10.3390/ijerph17114060

**Published:** 2020-06-06

**Authors:** Lara E. Tomenchok, Maribeth L. Gidley, Kristina D. Mena, Alesia C. Ferguson, Helena M. Solo-Gabriele

**Affiliations:** 1 Department of Civil, Architectural, and Environmental Engineering, University of Miami, Coral Gables, FL 33146, USA; let47@miami.edu; 2 Cooperative Institute for Marine and Atmospheric Studies (CIMAS), University of Miami, Miami, FL 33149, USA; maribeth.gidley@noaa.gov; 3 Atlantic Oceanographic and Meteorological Laboratory (AOML), National Oceanic and Atmospheric Administration (NOAA), Miami, FL 33149, USA; 4 School of Public Health, University of Texas, Houston, TX 77030, USA; Kristina.D.Mena@uth.tmc.edu; 5 Department of Built Environment, North Carolina A&T State University, Greensboro, NC 27411, USA; acferguson@ncat.edu

**Keywords:** abrasions, *Vibrio vulnificus*, children, wound infections, beach play

## Abstract

The Beach Exposure and Child Health Study (BEACHES) quantified the behavior of children at recreational beach areas to evaluate how various behaviors might affect their exposure to environmental contaminants such as bacteria and chemicals. Due to limited information in the study about abrasions, we conducted a literature review to examine how marine bacteria cause infections in open wounds. The literature review revealed possible adverse health effects from the bacterium *Vibrio vulnificus* due to its increasing prevalence and the severity of infection. We used data from the BEACHES study to review children’s behavior and their susceptibility to abrasions. Children six years of age and younger were evaluated before and after 1 h of play for open or healing abrasions at two beaches in Miami-Dade County, Florida (Crandon and Haulover), and two beaches in Galveston County, Texas (Stewart and Seawall). The children were videotaped to monitor their activities and to determine the behavior that would increase their susceptibility to obtaining abrasions. Overall, 58.2% of the children had at least one existing abrasion before playing at the beach, while 8.2% of the children acquired a new abrasion during their time at the beach. Children who acquired new abrasions most often played in the sea water, with new abrasions most frequently occurring on exposed skin surfaces such as the knees. Proper wound care before and after visiting the beach should be encouraged to minimize the risk of bacterial infection, especially considering the possible detrimental impacts that can be caused by some bacterial pathogens through wound exposures.

## 1. Introduction

In recent years, wound infections from recreational water sources have been reported at a seemingly higher rate than average. In July 2019, the Tampa Bay Times reported “Another case of flesh-eating bacteria sends Tarpon Bay fisherman to the hospital” [1]. Just one month later, the Miami Herald reported, “Man recovers from flesh-eating infection after Spring Break swim in a Florida river” [2]. These are just two examples of the many alarming cases reported in Florida in 2019. Group A streptococcus is the overall most common cause of flesh-destroying infections, referred to as necrotizing fasciitis. In the marine environment, however, the bacteria responsible for these severe infections include *Vibrio* spp., *Aeromonas* spp., *Shewanella* spp., and the halotolerant *Staphylococcus aureus*, with *Vibrio vulnificus* the most commonly identified species [3].

**Figure 1:**
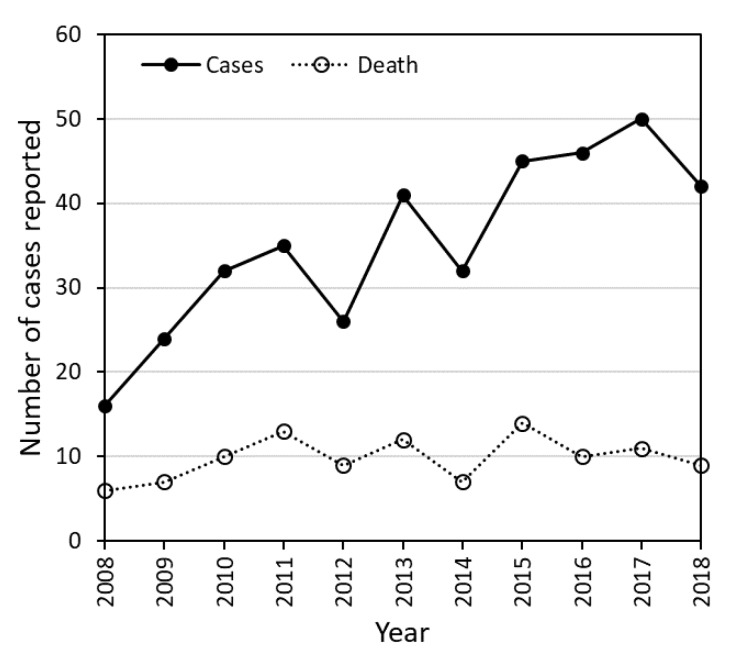
Number of confirmed cases and deaths of *V. vulnificus* in 32 Florida counties reported by the Florida Department of Health for 2008–2018.

Since 2008, the Florida Department of Health (FDOH) has reported an increasing number of *Vibrio vulnificus* cases (see Figure 1), stating that “*Vibrio vulnificus* is a rare cause of disease, but it is also underreported.” Because of the aggressive nature of these infections, the FDOH emphasizes that treatment should be initiated as soon as *V. vulnificus* is suspected [4]. Those most vulnerable to bacteria in water bodies include young children, the population above the age of 64, and those with chronic diseases or weakened immune systems [5]. Since children tend to contract wounds during play activities at the beach, their risk of exposure to *V. vulnificus* could be elevated.

The Beach Exposure and Child Health Study (BEACHES) quantified children’s behavior at recreational beach areas to evaluate how different types of behavior might affect their exposure to environmental contaminants [6]. Abrasions were defined as a compromised area of skin consisting of an open and bleeding wound, a slightly healed wound starting to scab, or a scratched wound with skin damage but not yet bleeding. To better evaluate the incidence of injuries at beaches, we collected lifeguard reports from the United States Lifesaving Association (USLA) for the two study areas, i.e., Miami-Dade and Galveston counties. Lifeguard reports for the past 5 years showed that about 1 in 15,000 people reported minor injuries in Miami-Dade County that required immediate care from a lifeguard, while about 1 in 5000 people reported minor injuries in Galveston County [7]. Several million people visit these beaches every year, and it is likely that the majority of injuries, especially minor abrasions, go unreported. Additionally, many people visit the beach with existing wounds that increase their risk of infection.

Infection risks are further increased due to the microbial landscape present at recreational beaches. The coincidence of wound injuries coupled with the microbial environment may increase the likelihood of wound infections. As such, documenting child abrasions and play behaviors that may increase exposure to infection is needed to evaluate risks. A supplemental literature review was conducted about how wound infections are acquired at beaches, and the proportion of children who had or acquired abrasions during play in a beach environment was evaluated on the basis of a study of 122 children. We are unaware of other studies that have evaluated children’s abrasions in beach settings.

### 1.1. Behavior

In Southern California, a study was conducted that analyzed marine bathing and associated health risks [8]. It was found that a paradox existed—most cases of illness associated with marine bathing arose from the lowest risk exposure (i.e., beach safe conditions), but higher numbers of people were exposed. One possible cause for this is that guidelines for beach closures are based solely on fecal indicator levels that do not include pathogen concentrations [9]. Consequently, large numbers of people may be exposed to pathogens that are unaccounted for in health advisories. Nonetheless, environmental bacteria species in seawater have been shown to cause wound infections regardless of whether the water was polluted with sewage [10]. A study by the Chicago Health, Environmental Exposure, and Recreation Department compared the health risks of limited-contact and full-contact activities. Contrary to general assumptions, the study found that there were health risks associated with limited-contact recreational activities in waterways, even when waters were designated as safe for swimming and other full-contact activities [11]. This finding indicates that people visiting recreational beach areas may be exposed to pathogens even when engaged in limited-contact activities.

The behavior of children at recreational beach areas may cause them to be more susceptible to contracting wounds with the potential to become infected. Minor injuries in children are common due to their developing physical ability, failure to recognize dangerous situations, and a willingness to robustly explore their environment [12]. A recent study found that children swim more often, stay in the water longer, submerge their heads more often, and swallow more water than adults while swimming [9]. Because wound infections are normally contracted during recreational activities such as swimming, fishing, or handling seafood, we must consider how children’s susceptibility to contracting wounds may expose them to pathogens such as *V. vulnificus*.

### 1.2. Pathogens

Many types of bacteria cause wound infections. The microorganisms that most notably present a risk include *V. vulnificus*, *Aeromonas* spp., and *Staphylococcus aureus*. For example, environmental surveys found that *V. vulnificus* was cultured from as much as 95% of estuarine samples collected in the northern part of Charlotte Harbor, Florida [13,14], and 45% of samples collected in Galveston Bay, Texas [15]. *Aeromonas* spp. was detected in 93% of freshwater lake samples [16] and found to be the cause of necrotizing fasciitis from contact with contaminated waters [17]. In Miami, *S. aureus* was isolated from 31% of ambient marine water samples and 25% of sand samples. Additionally, methicillin-resistant strains (MRSA) were isolated from samples collected from over half of the sampling days [18]. However, the most notorious bacteria in brackish and marine beach environments is *V. vulnificus*.

*V. vulnificus* is a Gram-negative aquatic bacterium that has been isolated from a range of environmental sources, including water, sediment, and seafood produce [19]. The infection can be contracted in two distinct ways: eating contaminated seafood or wound exposure [20]. *V. vulnificus* proliferates in low salinity waters and warmer temperatures, with the largest populations occurring in the summer [21,22]; thus, the highest incidences of *V. vulnificus* infection are reported during the summer months. Although ambient temperatures in tropical and subtropical regions enable the proliferation of *V. vulnificus*, cases have also been reported in temperate countries such as Denmark, Sweden, Spain, and Germany [23]. In colder regions, vibrios are common in areas of substantial terrestrial runoff, organic enrichment, and high summer water temperatures [10]. Additional factors that may affect *V. vulnificus* abundance and distribution are episodic storm events and riverine flow [24]. The Centers for Disease Control and Prevention (CDC) reported that the number of *V. vulnificus* infections increased by 54% in 2017 when compared with instances from 2014–2016 among 10 sites that account for 15% of the U.S. population [25]. This is concerning because *V. vulnificus* infections spread rapidly and aggressively and are associated with high mortality. Wound infection upon exposure often involves varying degrees of tissue damage, including cellulitis, septicemia, and soft-tissue infection, as well as fasciitis and gangrene [23,26,27]. One study using rats tested the risk of acquiring acute bacterial infections from beaches. It found that an average of 2.7 out of 3 rats with *V. vulnificus* infection died, with the average length of survival being only 16 h [10]. The Food and Drug Administration reviewed 459 cases in the United States from 1992 to 2007, revealing that there was a 51.6% mortality rate for individuals who acquired a *V. vulnificus* infection [27].

*V. vulnificus* is also responsible for 95% of seafood-related deaths in the United States [27] and has the highest mortality rate of any foodborne pathogen [28]. Because of this, it has been widely studied as a foodborne pathogen. Its role in wound infection is less studied, although approximately 25% of *V. vulnificus* infections are caused by direct exposure of open wounds to warm seawater containing the organism [29].

## 2. Materials and Methods

The objective of this study was to document the proportion of children with pre-existing abrasions, as well as the number of new abrasions children acquired while at the beach, and also to determine factors that may affect how wounds are obtained. After informed consent from guardians, we enrolled 122 children aged 6 years or younger (IRB# 20140140-MOD00023226 for the University of Miami and IRB# HSC-SPH-18-0396 for the University of Texas) in a study at one of four beaches—two in Miami-Dade County, Florida (Crandon or Haulover), and two in Galveston County, Texas (Stewart and Seawall). Seawall is also known as Urban Park Beach. Demographics were documented for each child that included their age and gender. Children were videotaped for 1 h to document the locations where they played, as well as their activity level and the objects and surfaces they contacted. Before and after their playtime, the children were checked for abrasions on exposed areas and areas reported by parents when asked about abrasions. The location of an abrasion was documented using a diagram representing the front, back, and side of each child. Insect bites were defined as singular raised areas on the skin. Insect bites were classified as abrasions if the skin was broken, slightly scabbed, or scratched with skin damage. During the abrasion check-in, a general question was asked about whether there were any concerns regarding the children playing at the beach. Very few guardians expressed concerns, and questions were typically raised about sunscreen, general weather conditions, and the ability to rinse off sand following the study. There was no evidence received about underlying health conditions that may increase the child’s risk of infection from wounds.

### Statistical Analysis

Data are publicly available through the Gulf of Mexico Research Initiative Information and Data Cooperative (GRIIDC) [30]. The data were used to compute basic statistics such as means and proportions of different subgroups. Binomial statistical distribution analyses were used to compute the significant differences and the 95% confidence intervals between different groups of data [31]. Odds ratios were used to evaluate the relation between the clothing worn by children and the number of new abrasions [32].

## 3. Results

**Figure 2:**
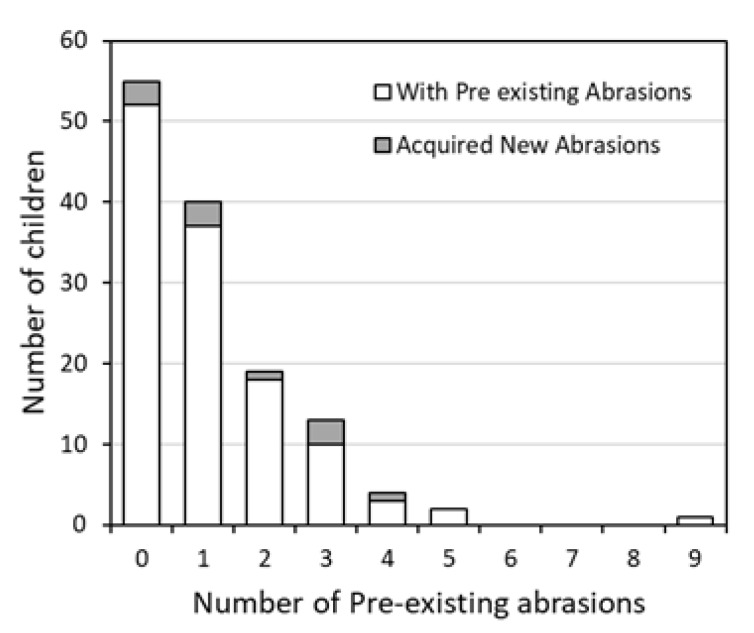
Number of children presenting with pre-existing abrasions compared to the number of children who acquired new abrasions during 1 h of beach play.

Of the 122 children, 71 had one or more pre-existing abrasions prior to the study. This corresponded to a total of 144 abrasions among the 71 children. Ten children acquired one or more new abrasions during their hour of play. The conditional relationships between the number of existing abrasions and the number of newly acquired abrasions were determined (see Figure 2, Table A1). The purpose of this was to observe whether a child with more pre-existing abrasions had behavioral tendencies that would make them more susceptible to obtaining new abrasions. For children arriving with no pre-existing abrasions, 3 out of 51 (confidence interval (CI) 0.012 to 0.162) acquired a new abrasion during the course of beach play. Similarly, among the children with one pre-existing abrasion, 2 out of 37 (CI 0.007 to 0.182) acquired a new abrasion. For children with two, three, and four pre-existing abrasions, 1 out of 18 (CI 0.001 to 0.273), 3 out of 10 (CI 0.067 to 0.653), and 1 out of 3 (CI 0.008 to 0.906), respectively, acquired a new abrasion. Children who arrived with five or more abrasions did not acquire additional abrasions during the study. Differences in proportions were statistically insignificant due to the increasing range of the confidence interval. This is because the total number of children who arrived with each number of existing abrasions decreased as the number of pre-existing abrasions increased.

For the 10 children who obtained a new abrasion, the ocean was the area most frequented, with five children spending most of their time in the water (see Table 1). This was followed by play in the intertidal area, with two children visiting this location the most. Following that, children spent most of their time in the dune ridge, with two children spending the majority of their time in this area. The last location that children frequently played was the berm crest, with only one child spending most of their time there. Play durations at other locations, including the sandbar, back beach, dune areas, and boardwalk, were less than 1% of the duration played on average. Children were not in view of the camera recording for the remaining portion of time.

In terms of gender, the ratio of males to females was 68:54. Of the children who acquired a new abrasion, 5 out of 10 were male and 5 out of 10 were female. Of the 71 children with pre-existing abrasions, 30 were male and 41 were female. The proportion of females versus males was statistically insignificant.

In terms of body part most susceptible to abrasions, we found that the majority of abrasions occurred on the legs, accounting for 61 of the 144 pre-existing abrasions (Table 2). The body parts with the second most abrasions were the arms and knees, with 23 of the 144 abrasions, respectively. The remaining parts of the body represented less than 10% of the total abrasions. For instance, eight occurred on the back, nine were on the head, eight were on the feet, five were on the front torso, five were on the hands, and two were on the elbows. Additionally, the conditional relationships between new abrasions and the body part showed that the proportion of new abrasions did not correspond to the body part of existing abrasions. For example, 61 of the 144 of existing original abrasions occurred on the legs, while only 2 of the 10 of the newly acquired abrasions were on the same body part. Likewise, only 9 of the 144 existing abrasions were on the head, whereas 2 of the 10 new abrasions occurred on the head.

The relationship between the body part of new abrasions and whether that body part was exposed was also investigated by evaluating odds ratios. Body part exposure was determined by the clothing the children wore during the experiment (e.g., if the child did not wear shoes, their feet were considered exposed, whereas if they did wear shoes, their feet were considered covered). Because only 10 children acquired new abrasions during the experiment, there was too small a sample size to identify significant trends at the 95% confidence interval. However, the evaluation of proportions suggests that if a child had a body part covered, the proportion of new abrasions was lower for that body part. For instance, all children who obtained new abrasions had legs and knees exposed, and 5 of the 10 of the abrasions occurred on these areas. All children had their heads exposed, and two new abrasions occurred on their heads. One reason more abrasions may not have occurred on the head is that it is a low contact area. One foot abrasion occurred on a child with exposed feet. Of the two children with new abrasions on their torso, one had their torso covered and the other did not. The odds ratio was calculated to be 7, but the confidence interval (0.22, 226) includes 1, and thus it is insignificant.

The type of abrasion each child received was also categorized into insect bites and non-insect bites. The majority of these bites were believed to be mosquito bites on the basis of visual observation (discrete erythematous) and parental identification. Of the children with abrasions (pre-existing and acquired during play), 33 of 74 had non-insect bites only, 23 had insect bites only, and 18 had both insect bites and non-insect bites. The type of abrasion for children at the Miami-Dade or Galveston county sites (see Table 3) was not statistically different at the 95% confidence level. For the children in Miami-Dade County, 20 out of 40 with abrasion(s) had non-insect bites only (CI 0.338 to 0.662), 9 had insect bites only (CI 0.108 to 0.385), and 11 had both non-insect and insect bites (CI 0.146 to 0.439). For the children in Galveston County, 13 out of 34 had non-insect bites only (CI 0.222 to 0.564), 14 had insect bites only (CI 0.247 to 0.593), and 7 had both non-insect bites and insect bites (CI 0.087 to 0.379).

## 4. Discussion and Recommendations

This is the first report that quantifies child abrasions in beach settings. This study found that pre-existing abrasions were observed in 58.2% of children before they played at the beach, and 8.2% of children acquired a new abrasion during one hour of play. These results suggest that a majority of children enter the beach environment with wounds that are susceptible to infection with a significant proportion of children attaining an additional wound during beach play. These results are significant in the context of the prevalence of pathogens within marine settings and speak to the importance of wound care for children before and after beach play activities, especially given the possible severity of wound infections such as those caused by *V. vulnificus*. Because beach closures are determined using fecal indicator bacteria (FIB) rather than pathogen levels, proper wound care should be practiced even in beach-safe conditions. One study found that *V. vulnificus* incidences were not correlated to fecal coliform or sewage contamination [33], and thus water that is considered safe for recreational activities may contain pathogens. Many reviews have compared the pros and cons of using FIB and have noted their failure to correlate with pathogens [34,35,36,37].

To prevent infection, parents should identify open abrasions and cover them with a waterproof bandage prior to allowing their child to play in a marine environment. Additionally, all wounds should be washed with clean water and soap both before and after play. New abrasions should be washed with clean water and soap immediately, then monitored closely for 1–2 days. A layer of antibiotic cream can also be applied. It is important for parents to monitor insect bites, as well as other types of wounds of the skin, because children have a higher tendency than adults to scratch aggressively. Additionally, since pre-existing insect bites can develop into a secondary infection, it is important for parents and physicians to recognize that different insect or spider bites may present with different appearances, and attempt to identify when necessary in order to carry out a prompt and appropriate treatment [38]. Of the 74 children who had abrasions, 55.4% had at least one insect bite which had been scratched. In a study evaluating the first aid incidents of children at New Zealand beaches, it was found that children 0–10 years received proportionally more insect stings than children 11–15 years [39]. Because younger children have a higher tendency to scratch insect related wounds, it is advised to apply insect repellent before playing outdoors.

Because marine water usually has detectable levels of wound-related pathogens, children should be closely monitored while playing in the water to encourage activities that minimize acquiring abrasions. At New Zealand beaches, it was found that children 0–5 years sustained 5% more of injuries while they were in the water compared to land based activities, and most injuries (55%) amongst children 0–16 years were sustained in water with swimming the most likely activity [39]. Furthermore, a study analyzing beach-related injuries in children identified rough/choppy water as an independent risk factor for beach injury [40]. Because the water was generally calm while this study was conducted, it is important to note that the risk associated with acquiring abrasions in the water is increased by these conditions.

Furthermore, in a study assessing microbes at a non-point source subtropical recreational marine beach, *Vibrio vulnificus* was detected in both sand and water samples [41]. A common behavior exhibited by young children is digging and burying themselves in the sand while sitting in shallow water. This puts them at a potentially greater dermal risk due to the detection of pathogens in sands [42,43,44] and because shallow water tends to have the most contaminants [45,46]. Thus, children’s play should be monitored both in water and on land to avoid the acquisition of abrasions, and existing wounds should be covered to avoid contact with sand.

To help prevent injury, parents may consider protective clothing for their children for areas that cover the most commonly abraded body parts. After one hour of play, it was found that most abrasions occurred on the legs, arms, and knees. Another study confirmed that the most common injury sustained by children at beaches was lacerations, and most occurred on the lower limbs [39]. Therefore, long sleeve swim shirts, swim pants, and protective shoes are good options for young children.

Generally, the public should avoid bathing in seawater following heavy rain, cover and clean all abrasions, shower with clean water before and after bathing, and wear clothing with more coverage. These measures will mitigate infection risks associated with exposure to wound-related pathogens. In addition, public education campaigns are needed to increase awareness of the incumbent risks in visiting recreational beach areas, especially for people with impaired immunity and open wounds.

## 5. Conclusions

We found that the majority of children had one or more preexisting abrasions before participating in one hour of beach play. Given the large proportion of susceptible children, future research is recommended for the evaluation of risks from open skin wounds. Skin wounds are susceptible to waterborne pathogens of non-fecal origin [34], pathogens in beach sand [45], and other possible contaminants at beaches [47,48]. More exposure data are needed to quantify and characterize skin wounds and to evaluate behaviors that may predispose children to contaminants during beach play. Further studies focused on children’s abrasions should allow for a longer period of play and involve a greater number of participants.

## Figures and Tables

**Table 1:** Duration of time and number of children at specified locations. Statistics correspond to the 10 children who acquired new abrasions.

Location	Average Duration of Time Spent at This Location Per Child (Seconds)	No. of Children Who Spent Most of Their Time at This Location
Seawater	1556	5
Intertidal	1069	2
Dune ridge	741	2
Berm crest	303	1
Sandbar	15.4	0
Back beach	6.4	0
Dune areas	2.8	0
Boardwalk	0.1	0
Not in view	2.9	0

**Table 2:** Body part most susceptible to abrasions.

Body Part where Abrasion Occurred	Total Number of Existing Abrasions	Total Number of New Abrasions	Amount Total Abrasions (%)
Hand	5	0	3.5%
Elbow	2	0	1.4%
Arm	23	0	16.0%
Knee	23	3	16.0%
Leg	61	2	42.4%
Feet	8	1	5.6%
Front torso	5	2	3.5%
Back	8	0	5.6%
Head	9	2	6.3%
Total	144	10	

**Table 3:** Type of abrasions broken down by county.

Type of Abrasion	Miami-Dade County			Galveston County		
	No. of Children with Abrasions	Percentage of Children with Abrasions (%)	Confidence Interval	No. of Children with Abrasions	Percentage of Children with Abrasions (%)	Confidence Interval
Non-insect only	20	50.0%	0.34 to 0.66	13	38.2%	0.22 to 0.56
Insect bites only	9	22.5%	0.11 to 0.39	14	41.2%	0.25 to 0.59
Both non-insect and insect bites	11	27.5%	0.15 to 0.44	7	20.6%	0.087 to 0.38
Total	40		Total	34		

## Appendix A

**Table A1:** Number of pre-existing abrasions per child with a comparison of the probability of acquiring a new abrasion during one hour of play.

Number of Pre-Existing Abrasions Before 1 Hour of Play	Percentage of Children Who Acquired New Abrasions During 1 Hour of Play (%)	Ratio of Children Who Acquired New Abrasions During 1 Hour of Play	Confidence Interval
0	5.8%	3/52	0.012 to 0.162
1	5.7%	3/37	0.007 to 0.182
2	5.3%	1/18	0.001 to 0.273
3	30.0%	3/10	0.067 to 0.653
4	33.3%	1/3	0.008 to 0.906
5	0	0/2	N/A ^a^
6	0	0/0	N/A
7	0	0/0	N/A
8	0	0/0	N/A
9	0	0/1	N/A

^a^ N/A = not applicable.

## References

[1] Griffin J. Another Case of Flesh-Eating Bacteria Sends Tarpon Springs Fisherman to the Hospital. Tampa Bay Times. https://www.tampabay.com/health/another-case-of-flesh-eating-bacteria-sends-tarpon-springs-fisherman-to-the-hospital-20190731/.

[2] Ocasio B.P. Man Recovers from Flesh-Eating Infection after Spring Break Swim in a Florida River. Miami Herald. https://www.miamiherald.com/living/health-fitness/article233522322.html.

[3] Park K.H., Hwang J.H., Jung S.I., Shin J.H., Jung Y.S. (2009). Marine bacteria as a leading cause of necrotizing fasciitis in coastal areas of South Korea. Am. J. Trop. Med. Hyg..

[4] Florida Department of Health Vibrio Infections. http://www.floridahealth.gov/diseases-and-conditions/vibrio-infections/vibrio-vulnificus/index.html.

[5] Florida Department of Health What You Need to Know about Necrotising Fasciitis; Commonly Called “Flesh-Eating Bacteria.” Florida Department of Health in Hillsborough County. http://hillsborough.floridahealth.gov/newsroom/2019/07/NECROTIZING_FASCIITIS.html.

[6] Ferguson A., Del Donno C., Obeng-Gyasi E., Mena K., Kaur Altomare T., Guerrero R., Gidley M., Montas L., Solo-Gabriele H.M. (2019). Children exposure behavior patterns and risk perception with recreational beach use. Int. J. Env. Res. Public Health.

[7] United States Lifesaving Association (USLA) USLA—Statistics. http://arc.usla.org/Statistics/public.asp.

[8] Brinks M.V., Dwight R.H., Osgood N.D., Sharavanakumar G., Turbow D.J., El-Gohary M., Caplan J.S., Semenza J.C. (2008). Health risk of bathing in Southern California coastal waters. Arch. Environ. Occup. Health.

[9] Schets F.M., Schijven J.F., Husman A.M.D.R. (2011). Exposure assessment for swimmers in bathing waters and swimming pools. Water Res..

[10] Kueh C., Kutarski P., Brunton M. (1992). Contaminated marine wounds-the risk of acquiring acute bacterial infection from marine recreational beaches. J. Appl. Bacteriol..

[11] Holtcamp W. (2012). In the same boat? Health risks of water recreation are not limited to full-contact activities. Environ. Health Perspect..

[12] Young S.J., Barnett P.L.J., Oakley E.A. (2005). 10. Bruising, abrasions and lacerations: Minor injuries in children I. Med. J. Aust..

[13] Lipp E.K., Kurz R., Vincent R., Rodriguez-Palacios C., Farrah S.R., Rose J.B. (2001). The effects of seasonal variability and weather on microbial fecal pollution and enteric pathogens in a subtropical estuary. Estuaries.

[14] Lipp E.K., Rodriguez-Palacios C., Rose J.B. (2001). Occurrence and distribution of the human pathogen *Vibrio vulnificus* in a subtropical Gulf of Mexico estuary. Ecol. Etiol. New. Emerg. Mar. Dis..

[15] Lin M., Payne D.A., Schwarz J.R. (2003). Intraspecific diversity of *Vibrio vulnificus* in Galveston Bay water and oysters as determined by randomly amplified polymorphic DNA PCR. Appl. Environ. Microbiol..

[16] Mathai P.P., Dunn H.M., Magnone P., Zhang Q., Ishii S., Chun C.L., Sadowsky M.J. (2019). Association between submerged aquatic vegetation and elevated levels of *Escherichia coli* and potential bacterial pathogens in freshwater lakes. Sci. Total Environ..

[17] Fernández-Bravo A., Kilgore P.B., Andersson J.A., Blears E., Figueras M.J., Hasan N.A., Colwell R.R., Sha J., Chopra A.K. (2019). T6SS and ExoA of flesh-eating *Aeromonas*
*hydrophila* in peritonitis and necrotizing fasciitis during mono- and polymicrobial infections. Proc. Natl. Acad. Sci. USA.

[18] Plano L.R.W., Shibata T., Garza A.C., Kish J., Fleisher J., Sinigalliano C.D., Gidley M.L., Withum K., Elmir S.M., Hower S. (2013). Human-associated methicillin-resistant *Staphylococcus aureus* from a subtropical recreational marine beach. Microb Ecol..

[19] Baker-Austin C., Oliver J.D. (2017). *Vibrio vulnificus*: New insights into a deadly opportunistic pathogen. Environ. Microbiol..

[20] Shaw K.S., Sapkota A.R., Jacobs J.M., He X., Crump B.C. (2015). Recreational swimmers exposure to *Vibrio vulnificus* and *Vibrio parahaemolyticus* in the Chesapeake Bay, Maryland, USA. Environ. Int..

[21] Kelly M.T. (1982). Effect of temperature and salinity on *Vibrio* (Beneckea) *vulnificus* occurrence in a gulf coast environment. Appl. Environ. Microbiol..

[22] Nigro O.D., Hou A., Vithanage G., Fujioka R.S., Steward G.F. (2011). Temporal and spatial variability in culturable pathogenic Vibrio spp. in Lake Pontchartrain, Louisiana, following Hurricanes Katrina and Rita. Appl. Environ. Microbiol..

[23] Oliver J.D. (2005). Wound infections caused by *Vibrio vulnificus* and other marine bacteria. Epidemiol. Infect..

[24] Wetz J., Blackwood A., Fries J., Williams Z., Noble R. (2008). Trends in total *Vibrio* spp. and *Vibrio vulnificus* concentrations in the eutrophic Neuse River Estuary, North Carolina, during storm events. Aquat. Microb. Ecol..

[25] (2018). Centers for Disease Control and Prevention (CDC). Preliminary Incidence and Trends of Infections with Pathogens Transmitted Commonly through Food—Foodborne Diseases Active Surveillance Network, 10 U.S. Sites, 2006–2017. Morb. Mortal. Wkly. Rep..

[26] Horseman M.A., Surani S. (2011). A comprehensive review of *Vibrio vulnificus*: An important cause of severe sepsis and skin and soft-tissue infection. Int. J. Infect. Dis..

[27] Jones M.K., Oliver J.D. (2009). *Vibrio vulnificus*: Disease and pathogenesis. Infect. Immun..

[28] Rippey S.R. (1994). Infectious diseases associated with molluscan shellfish consumption. Clin. Microbiol. Rev..

[29] Bross M.H., Soch K., Morales R., Mitchell R.B. (2007). Vibrio vulnificus Infection: Diagnosis and treatment. Am. Fam. Physician.

[30] Gulf Research Initiative (GRI). https://data.gulfresearchinitiative.org.

[31] Pezzullo J.C. (2019). Exact Binomial and Poisson Confidence Interval. Stat Pages. http://statpages.info/confint.html.

[32] Select Statistical Services (SSS) Odds Ratio—Confidence Interval. https://select-statistics.co.uk/calculators/confidence-interval-calculator-odds-ratio/.

[33] Riviera S., Lugo T., Hazen T.C. (1989). Autecology of *Vibrio vulnificus* and *Vibrio parahaemolyticus* in tropical waters. Wat. Res..

[34] Boehm A.B., Ashbolt N.J., Colford J.M., Dunbar L.E., Gold M.A., Hansel J.A., Hunter P.R., Ichida A.M., McGee C.D., Soller J.A. (2009). A sea change ahead for recreational water quality criteria. J. Water Health.

[35] Field K.G., Samadpour M. (2007). Fecal source tracking, the indicator paradigm, and managing water quality. Water Res..

[36] Hardwood V.J., Staley C., Badgley B.D., Borges K., Korajkic A. (2014). Microbial source tracking markers for detection of fecal contamination in environmental waters: Relationships between pathogens and human health outcomes. FEMS Microbiol..

[37] Korajkic A., McMinn B.R., Harwood V.J. (2018). Relationships between microbial indicators and pathogens in recreational water settings. Int. J. Env. Res. Public Health.

[38] Paolino G., Vaira F., Mercuri S.R., Di Nicola M.R. (2020). Fast recognition of Loxosceles rufescens in Italian spider bites to avoid misdiagnosis, alarmism and start a prompt treatment. J. Eur. Acad. Derm. Venereol..

[39] Moran K., Webber J. (2014). Surf, sand, scrapes and stings: First aid incidents involving children at New Zealand beaches, 2007–2012. J. Paediatr. Child Health.

[40] Petronis K.A., Welch J.C., Pruitt C.W. (2009). Independent risk factors for beach-related injuries in children. Clin. Pediatrics.

[41] Abdelzaher A.M., Wright M.E., Ortega C., Hasan A.R., Shibata T., Solo-Gabriele H.M., Kish J., Withum K., He G., Elmir S.M. (2011). Daily Measures of Microbes and Human Health at a Non-point Source Marine Beach. J. Water Health.

[42] Whitman R.L., Harwood V.J., Edge T.A., Nevers M.B., Byappanahalli M., Vijayavel K., Brandão J., Sadowsky M.J., Wheeler Alm E., Crowe A. (2014). Microbes in beach sands: Integrating environment, ecology and public health. Rev. Environ. Sci. Biotechnol..

[43] Solo-Gabriele H.M., Harwood V.J., Kay D., Fujioka R.S., Sadowsky M.J., Whitman R.L., Wither A., Caniça M., Carvalho da Fonseca R., Duarte A. (2016). Beach Sand and the Potential for Infectious Disease Transmission: Observations and Recommendations. J. Mar. Biol. Assoc. UK.

[44] Weiskerger C.J., Brandão J., Ahmed W., Aslan A., Avolio L., Badgley B.D., Boehm A.B., Edge T.A., Fleisher J.M., Heaney C.D. (2019). Impacts of a changing earth on microbial dynamics and human health risks in the continuum between beach water and sand. Water Res..

[45] Shah A.H., AbdelZaher A.M., Phillips M., Hernández R., Solo-Gabriele H.M., Kish J., Fleming L.E. (2012). Indicator microbes correlate with pathogenic bacteria, yeasts and helminthes in sand at a subtropical recreational beach site. J. Expo. Sci. Technol..

[46] Wright M.E., AbdelZaher A.M., Solo-Gabriele H.M., Elmir S., Fleming L.E. (2011). The intertidal zone is the pathway of input of enterococci to a subtropical recreational marine beach. Water Sci. Technol..

[47] Black J.C., Welday J.N., Buckley B., Ferguson A., Gurian P.L., Mena K.D., Yang I., McCandlish E., Solo-Gabriele H.M. (2016). Risk assessment for children exposed to beach sands impacted by oil spill chemicals. Int. J. Environ. Res. Public Health.

[48] Ferguson A.C., Mena K.D., Solo-Gabriele H.M. (2020). Assessment for oil spill chemicals: Current knowledge, data gaps and uncertainties addressing human physical health risk. Mar. Pollut. Bull..

